# The Health Opportunity Index: Understanding the Input to Disparate Health Outcomes in Vulnerable and High-Risk Census Tracts

**DOI:** 10.3390/ijerph17165767

**Published:** 2020-08-10

**Authors:** Chinonso N. Ogojiaku, JC Allen, Rexford Anson-Dwamena, Kierra S. Barnett, Olorunfemi Adetona, Wansoo Im, Darryl B. Hood

**Affiliations:** 1 Division of Environmental Health Sciences, College of Public Health, Ohio State University, 408 Cunz Hall, 1841 Neil Ave., Columbus, OH 43210, USA; ogojiaku.1@osu.edu (C.N.O.); adetona.1@osu.edu (O.A.); 2 Office of Health Equity, Ohio Department of Health, Columbus, OH 43215, USA; Chip.Allen@odh.ohio.gov; 3 Office of Health Equity, Virginia Department of Health, Richmond, VA 23219, USA; Rexford.Dwamena@vdh.virginia.gov; 4 The Kirwan Institute for the Study of Race and Ethnicity, Ohio State University, Columbus, OH 43201, USA; barnett.433@osu.edu; 5 Division of Public Health, Meharry Medical College, Nashville, TN 37208, USA; wim@mmc.edu

**Keywords:** health opportunity index, health equity, health disparities, social determinants of health, principal component analysis, GIS, Ohio, thematic mapping, disease convergence, public health exposome

## Abstract

The Health Opportunity Index (HOI) is a multivariate tool that can be more efficiently used to identify and understand the interplay of complex social determinants of health (SDH) at the census tract level that influences the ability to achieve optimal health. The derivation of the HOI utilizes the data-reduction technique of principal component analysis to determine the impact of SDH on optimal health at lower census geographies. In the midst of persistent health disparities and the present COVID-19 pandemic, we demonstrate the potential utility of using 13-input variables to derive a composite metric of health (HOI) score as a means to assist in the identification of the most vulnerable communities during the current pandemic. Using GIS mapping technology, health opportunity indices were layered by counties in Ohio to highlight differences by census tract. Collectively we demonstrate that our HOI framework, principal component analysis and convergence analysis methodology coalesce to provide results supporting the utility of this framework in the three largest counties in Ohio: Franklin (Columbus), Cuyahoga (Cleveland), and Hamilton (Cincinnati). The results in this study identified census tracts that were also synonymous with communities that were at risk for disparate COVID-19 related health outcomes. In this regard, convergence analyses facilitated identification of census tracts where different disparate health outcomes co-exist at the worst levels. Our results suggest that effective use of the HOI composite score and subcomponent scores to identify specific SDH can guide mitigation/intervention practices, thus creating the potential for better targeting of mitigation and intervention strategies for vulnerable communities, such as during the current pandemic.

## 1. Introduction

According to Healthy People 2020, Social Determinants of Health (SDH) are conditions in the environments in which people are born, live, learn, work, play, and pray that affect a wide range of health, functioning, and quality-of-life outcomes and risks [1]. A shift in focus in the field of public health has caused additional attention to be paid to SDH. Understanding the impact these determinants have on a community’s health is a vital part of achieving health equity [2]. Researchers estimate that approximately 40% of health can be attributed to social and economic factors and 10% are due to factors related to the physical environment [3]. These SDH vary both between and within communities based on variety of demographic characteristics. Examples of SDH include access to healthcare services, neighborhood crime and violence levels, and food security and availability.

Due to the current coronavirus disease 2019 (COVID-19) pandemic, the United States is currently experiencing a higher number in cases and deaths than what we are seeing globally [4]. The asymptomatic nature of the disease combined with pattern of transmission of COVID-19, emphasizes the importance of community health. Within the U.S., there are existing disparities of underlying health conditions that influences the way the pandemic is impacting marginalized groups and communities. The disparities in COVID-19 related health outcomes can be linked to a number of factors. African American communities and other communities of color in New York for example, are experiencing higher rates of infection and death during the COVID-19 pandemic than their white counterparts within the city [5]. Various factors within these communities render them to being more vulnerable to disease. For example, residents are struggling to access healthcare resources in their neighborhoods, while multigenerational homes put older residents’ home at risk. In addition, communities that have higher perveances of heart disease, diabetes, and other chronic diseases are seeing worse outcomes. Furthermore, the composition of the essential worker population is predominantly black and brown, who do not have the luxury of staying at home. This results in these workers returning to their segregated community with the higher potential of spreading infectious diseases. Public health practitioners, health systems, and community-based organizations often do not have tools to understand the complex interaction of social determinants linked to poor health outcomes. Given that disparate health outcomes from chronic conditions or pandemics such as COVID-19 are influenced by SDH and underlying health conditions, the ability to target these high-risk communities and provide interventions with a high degree of precision is critical to slowing the spread of the pandemic.

Health Opportunity Index (HOI) is a tool to understand the effect of SDH on health outcomes. Tools such as the HOI can demonstrate how the SDH can shape a community’s overall opportunity for optimal health. Using tools for geospatial analysis with data from the HOI is an effective method of identifying health disparities as well as the SDH, which drive these disparities out of control [6]. A thematic map can overlay SDH over a community of interest, highlighting rates of a determinant or adverse health outcome in the area. While such an approach is functional—a more robust, informative method needs to be explored in order to develop a deeper understanding of a community’s health. The HOI is a tool used to better identify the determinants in a community that drive poor health outcomes. The utilization of the HOI can assist in identifying communities with the lowest health outcomes and reduce health disparities.

The HOI was developed by Rexford Anson-Dwamena, an epidemiologist and GIS/spatial analyst at Virginia’s Public Health Department. The tool was later adopted by Johnnie (Chip) Allen, Director of Health Equity at the Ohio Department of Health (ODH). Allen found that the HOI could become a useful asset in the state’s effort to reduce health disparities and actively pursue true health equity. Health equality calls for providing the same health interventions to all individuals, regardless of the level of health disparity they face. Health equity, on the other hand, is achieved when every individual has the same opportunity to achieve their full health potential. Achieving health equity requires the identification of communities that might need a variety of interventions to overcome social, economic, and structural barriers to reach their peak level of health. SDHs are multifaceted, complex and often operate at varying levels of severity from one neighborhood to the next. The dynamic nature of SDH makes it difficult to address them in a comprehensive manner. Moreover, tools required for understanding the dynamic nature of SDH are needed now more than ever. One of ODH’s primary equity goals is to develop wide-spread capacity to identify and understand how specific SDHs operate at the local/neighborhood level. This understanding can empower local communities to comprehensively address these issues using targeted interventions with the help of broad-based coalitions.

The HOI utilizes principal component analysis in order to create an individual score for each census tract. Principal component analysis (PCA) is a statistical procedure used in exploratory data analysis. PCA is useful for data reduction and simplification, modeling, outlier detection, variable selection, classification, predictions, and unmixing. For the HOI, PCA’s data reduction functionality is critical. For example, literacy and educational determinants are both SDH and are strongly correlated. Food accessibility and hunger behave similarly. For this reason, it can be useful to adopt clustering methodology approaches in an attempt to identify SDH within census tract profiles. Common profiles include economic stability, education, health and built environment. While the HOI is a relatively new tool to the public health field, it shares similarities with another tool. The Child Opportunity Index (COI) is a tool that measures the resources and conditions of a neighborhood that are critical to the development of a child [7]. Its purpose is similar to the HOI, in that the tool aims to compare opportunity levels of different neighborhoods around the country under one metric.

Therefore, the goals of the present analyses are to; (1) educate environmental public health professionals and communities about the conditions children are exposed to in their neighborhoods, (2) inform the relevant policy makers who control the distribution of resources, and (3) improve equity and health outcomes in vulnerable populations. The index uses 29 indicators to measure neighborhood health for residents. Some of these indicators include school poverty, access to healthy food, access to green space, home ownership rate as well as other related indicators. Each indicator has an individual weight which correlates to the indicators ability to impact health. The indicators are all measured on different scales. For example, high school graduation rate and employment rate are expressed using percentages versus the hazardous waste site index, which is measured using a count. In order to combine these values, the raw data is standardized using z-score transformation. These indicators are then combined into one of three domains: education, health and environment, and social and economic. The scores are then weighted based on their health and economic impact and combined to a single score, scaled between 0 and 100. The COI contains information on over 72,000 census tracts that can be compared with other metro areas and across time periods. The COI 1.0 was released in 2014 and version 2.0 was released in January 2020.

Disparate health outcomes do not exist in a vacuum. Moreover, it is important but difficult to understand how disparate health outcomes simultaneously exist at their worst levels and the social determinants which impact these disparities. To help understand how these issues are connected, J. Chip Allen also developed the Convergence Analysis (CA) to help contextualize the HOI. The CA is a technique that uses geographically referenced health data with GIS mapping tools to visualize where various disparate health outcomes simultaneously exist at their worst levels. Any dataset that is geographically referenced can be used with the CA. This paper reflects the CA which uses selected data sets from the CDC 2017 Behavior Risk Factor Surveillance Survey (BRFSS) 500 Cities Project, 2013–2017, Ohio Department of Health Vital Statistics Data on prematurity and as well as 2017 data on lead exposure from the Ohio Department of Health at the census tract level. Specific datasets for the 2017 BRFSS 500 Cities Project data included crude prevalence rates of asthma, coronary heart disease, diabetes, high blood pressure, stroke, and poor mental health.

## 2. Methodology

The Health Opportunity Index (HOI) is the primary outcome variable in this study and is comprised of 13 indices: affordability, income inequality, Townsend Deprivation, job participation, employment access, education, population churning, population-weighted density, segregation, food accessibility, walkability, access to care, and environmental quality index. These 13 indices are separated into profiles based on how they co-exist in the state of Ohio. Indices will fall into the environmental, consumer, mobility, or economic profile. HOI scores are calculated for each census tracts in order to highlight specific community needs. Scores of census tracts were converted into shapefiles, then uploaded to ArcGIS to be displayed over a thematic map. HOI scores ranged from 0 to 1. The closer to zero the score is, the lower the probability residents in the census tract have the opportunity to achieve optimal health. A score closer to 1 signifies greater opportunity to achieve good health. The following section describes the methodology used to create the 13-index scores that make up the HOI within each of the four profiles.

The Environmental Profile consists of six indices, which include affordability, health care access, walkability, employment, population density, and environmental quality. Each index is described below.

The Affordability Index measures the proportion of a neighborhood’s income that is spent on housing and transportation. The equation used to derive the affordability index is as follows:

(1) $$\mathrm{Affordability}\;\mathrm{Index}=\frac{\mathrm{Housing}\;\mathrm{Cos}t+\mathrm{Transportation}\;\mathrm{Cos}t}{\mathrm{Total}\;\mathrm{Income}}.$$

The US Census Bureau has its own metric that it uses to measure poverty. However, this metric carries its own flaws as it assumes that the cost-of-living is uniform across the United States. This assumption is known to be false. To better account for this, the Affordability Index of the HOI identifies the most significant expenses a family incurs and weights the impact of that cost on disposable income [8]. The two major expenses factored into affordability are housing and transportation. The proportion of income spent on these two combined factors help account for the cost-of living variability and provide a better sense of affordability.

The Health Care Access index measures an area’s access to healthcare. The is accomplished using a two-step process to assess physician availability [9]. First, within a predetermined travel radius from a healthcare provider, the total population that the supplier could reach is established. This is then converted to a provider-to population ratio, with the total number of providers in a healthcare setting being accounted for. Next, once the area of interest (census tract) is established, the same predetermined travel radius is applied. Every healthcare setting in the radius is captured and the provider-to-population ratios are summed to give part of the access to care index. The additional portion of the index comes from the percentage of uninsured population. The percentages come from the American Community Survey, which records uninsured levels at the census tract level.

The Walkability Index is an EPA tool used to differentiate Census groups based on their walkability. The walkability of an area is based on the built environment that promotes or discourages walking as a mode of transportation. Factors such as street connectivity, crime, and facilities for walking are examples of factors that can contribute to the walkability of an area [10]. The walkability index is derived from a four-variable formula from the International Physical Activity and the Environment Network (IPEN). The equation is as follows:

(2) WAI=(2×con)+ent+far+hdens.

The four variables represent what is known as the 4D’s. Design (2 × con), diversity (ent), distance (far), and density (hdens) make up the new walkability index. Design refers to the design of the built environment and safety features of an area. A large number of street crossings that support safe pedestrian travel in a census block will increase this variable. Diversity refers to the diversity of land use in the census block. It measures the variety of activities that are within a walkable distance. Distance measures the distance to transit or how accessible it is for pedestrians to reach a transit stop. Density refers to residential and employment density. These variables measure the density of activities within a walkable distance.

The Employment Access Index measures the accessibility of jobs in a particular area. Poor job access can include barriers such as distance from residents and transportation availability. Communities that experience poor employment access tend to have more employment instability [11], which in turn leads to more socioeconomic disadvantages. The employment access index score is determined by utilizing a gravity model. The equation for the index is as follows:

(3) $$A_{i}=\sum_{j=1}^{n}\frac{J_{i}d_{ij}^{-\beta }}{V_{j}},\;where\;V_{j}=\sum_{k=1}^{m}W_{k}d_{kj}^{-\beta },$$

*A_i_* = job accessibility at location *i,*

*J_j_* = the number jobs in location *i,*

*d* = the travel time between them,

*β* = the friction coefficient,

*n* = the total number of job locations,

*V_j_* = job location’s proximity to all workers.

This index is calculated by summing the total number of jobs in an area and dividing by the square of the distance to those jobs. This allows for further examination of the presence and proximity of jobs in an area. The gravity model makes it possible to observe what job opportunities are available in or around block group or census tract, which provides a better picture of job access.

The Population-weighted Density Index is critical to better understand the spatial differences between rural and urban populations [12]. The index captures the density at which the average individual lives. The index is calculated by dividing the total population-weighted by the square miles in the area. The higher the index score, the higher the concentration of people per square mile.

The Environmental Quality Index is calculated using the EPA’s National Air Toxics Assessments (NATA) environmental data. This assesses the level of air pollution by census tract. The NATA has six indicators: neurological risk, cancer risk, respiration risk, on-road pollution, non-road pollution, and non-point pollution. The six indicators are combined into one indicator using principal component analysis. Principal component analysis converts the individual variables into latent variables which are then standardized into hazard quotients. Higher hazard quotients result in a higher possibility that the exposure to environmental conditions will result in a negative health outcome.

The Consumer Profile consists of three indices which include education, food access, and material deprivation. These indices are described below.

The Education Index measures the average education level achieved by the adult population in an area [13]. This is based on the average years of schooling in an area. Higher educational attainment has been shown to have positive impacts on economic earning and positive health outcomes. The equation for this index is as follows:

(4) $$\mu =AYS=\overset{\;n}{\underset{i=1}{\sum }}p_{i}y_{i},$$

*AYS* = average years of schooling,

*μ* = average years of schooling for the concerned population,

*p_i_* = proportion of population with certain level of schooling,

*y_i_* = years of schooling at different education attainment levels.

This index however does not take into account the quality of the education received, only average level of attainment.

The Food Accessibility Index measures low access to grocery stores, supermarket, and other suppliers of healthy foods. Proximity to healthy food options plays a critical role in maintaining a well-balanced and healthy diet [14]. The index score is based on the proportion of the census tract population that fits into one of three USDA criteria. The criteria on whether a significant portion of the population (500 individuals) or 33% of a low-income census tract live within a certain proximity to a grocery store or supermarket. The proximity to grocery store or supermarket depends on the population density of the census tract. Residents who live further than 0.5 or 1 mile away from a grocery store or supermarket in an urban area are considered to have low food access. Residents who live further than 10 or 20 miles away from a grocery store or supermarket in a rural area are considered to have low food access.

The Townsend Deprivation Index is used to measure material deprivation. Material deprivation, according to sociologist Peter Townsend, encompasses the lack of goods, services, amenities, resources and physical environment that are typically found and approved by society [15]. The index is made up of four variables: unemployment, car ownership, home ownership, and overcrowding. The unemployment variable is derived by determining the percentage of active residents in a community between the ages of 16 to 64 who are unemployed. The car ownership variable is based on the percentage of private households who do not possess a car. Home ownership is based off the percentage of private homes not currently occupied by the homeowner. The overcrowding variable is the percentage of private households with more than one person per room. Each of these variables are equally weighed and combined together to determine the Townsend deprivation index. In order to combine the variables, first the unemployment variable and the overcrowding variable must be log transformed. Next, the z-score is calculated for each of the four variables and the scores are summed up for the composite score for the index. The higher the score, the more a particular area lacks access to the resources.

The third profile in the HOI is the Economic Profile, which consists of three indices: job participation, segregation, and income inequality, which are described below:

The Job Participation index is a measure of the percentage of working aged individuals (16–64 years of age) in the active labor force. The equation for the variable is as follows

(5) $$\mathrm{Job}\;\mathrm{Participation}\;\mathrm{rate}=\frac{\mathrm{Civilian}\;\mathrm{Employed}+\mathrm{Civilian}\;\mathrm{Unemployed}}{\mathrm{Civilian}\;\mathrm{Population}\;\left(16-64\;\mathrm{yrs}\right)}.$$

Unlike the unemployment variable of the Townsend deprivation index, the job participation index is supposed to identify the employment rate of the active working class of an area. This index is considered very sensitive to the attributes of the local community. Attributes such as educational attainment, household composition, and car ownership can all influence an area’s employment rates. This index is a strong indicator of economic growth and income, which are strong factors in individual and community health status [16]. The higher the index, the healthier the labor market.

The Segregation Index, or the spatial dissimilarity index, measures how the racial composition of a population in a census tract compares to that of the rest of the state [17]. The mapping software tool ArcGIS (MapplerX, Nashville, TN, USA) has a toolbox to assist in the calculation of this index. The equation to calculate spatial dissimilarity is noted below.

(6) $$\begin{array}{c} SD=\left(\frac{1}{2}\right)(\sum_{i=1}^{n}\sum_{j=1}^{m}\left|CN_{i}-CE_{i}\right|i\sum_{j=1}^{m}CN\times CP_{j}(\left(1-CP_{j}\right)\;with\;\;CE_{i}\\ =\frac{\left(CN_{i}-CN_{j}\right)}{CN}\;and\;CN_{ij}=\sum_{k=1}^{n}d\left(N_{ij}\right), \end{array}$$

*CN_ij_*: Composite population count of ethnic group *j* in spatial unit *i,*

*d*(_ij_): Function defining surrounding spatial units *I* and *k,*

*CN_i_*: Total composite population count in spatial unit 1,

*CN_j_*: Total composite population count of ethnic group *j,*

*CN*: Total population in the city,

*CP_j_*: Proportion of population in ethnic group *j.*

The Income Inequality Index within the HOI is measured using the Gini coefficient. The Gini coefficient (index) is a statistical measure typically used to measure economic inequality [18]. The coefficient accomplishes this by measuring the diversity of actual earned income of a neighborhood. The equation is as follows:

(7) $$GINI=\frac{1}{\mu N\left(N-1\right)}\underset{i>j}{\sum }\underset{j}{\sum }\left|y_{i}-y_{j}\right|,$$

*μ* = the mean of the variable (income),

*N* = total number of observations,

*y_i_* and *y_j_* = dollar values of income of individuals.

Absolute neighborhood equality, or homogeneity, would result in a Gini coefficient of 0. Complete diversity in income for a neighborhood would result in a Gini coefficient of 1. Income inequality is a critical variable to account for due to its correlation with health outcomes. As the wage gap in the United States continues to increase, the disparities in health outcomes and life expectancy between high-income and low-income Americans continue to increase [19].

The final profile in the HOI is the Population Mobility Profile, which includes the population churning index and is described below.

The Churning Index is used to capture the total migration of individuals into a community. Population churning can bring both positive and negative aspects to a community. The in migration can bring an influx of social capital and opportunities. New community member can bring new business and employment opportunities. On the other hand, out migration can lead to the disruption of services that are critical to health and wellness [20]. The equation for the population churning index is as follows:

(8) $$\mathrm{Population}\;\mathrm{Churning}\;\mathrm{Rate}=\frac{\mathrm{In}\;\mathrm{migration}+\mathrm{out}\;\mathrm{migration}}{\mathrm{Total}\;\mathrm{population}}.$$

Population churning accounts for population movement in a manner that is different from net migration. Rather than simply indicating the balance of movement as net migration does, population churning gives a standardized measure of the amount of movement in relation to the population at large. For the HOI, census mobility data was used to show 5-year mobility patterns.

## 3. Quantitative Validity and Reliability of the HOI as an Assessment/Predictive Tool for Health Outcomes

**Figure 1:**
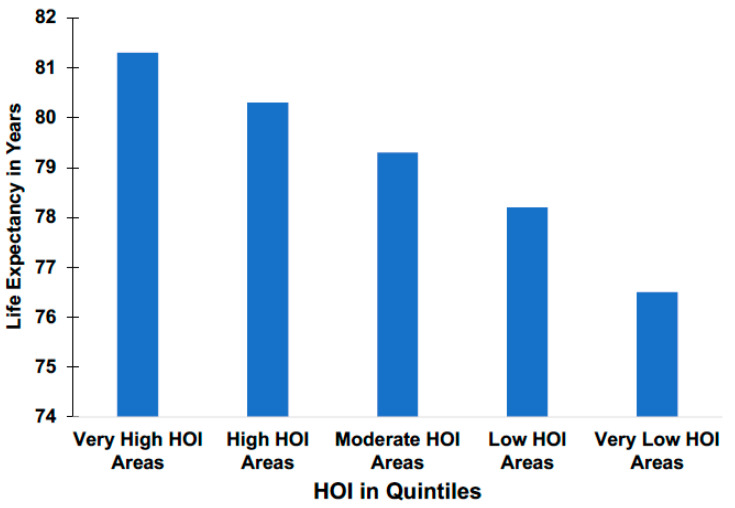
Health Opportunity Index Scaled into Quintiles against life expectancy to show the relationship. The relationship is monotonic indicating that persons living in “Very High Opportunity Areas” on average is expected to live over four more years compared to the persons in “Very Low Opportunity Areas”.

To provide assurance that the HOI was truly sensitive to major shifts in health status throughout the state, we used the most general definitions of good health, i.e., “life expectancy at birth” (LE) and “years of potential life lost” (YPLL) data to determine if the HOI demonstrated systematic public health differences between areas. Figure 1, above present’s life expectancies at birth by HOI quintiles. The indicators (LE and YPLL) were chosen as the health outcomes of interest because they; (1) provide a global indication of health across a population and across the life span; and (2) summarize health in a manner that is easily understood by all—how long a life can I expect to live? Our indicators also aligned with the notion that social determinants of health (SDH) are fundamental causes of disease. Therefore, access or lack of access to SDH would be expected to influence the entire range of health outcomes, and not just those associated with the same behavior (for example, physical activity being associated with obesity and diabetes but not HIV).

HOI was constructed to simplify a very complex social landscape important to the health of a community by specifying some very simple process-oriented indicators and determine how they interact within a local spatial context. Of course, no traditional public health concern (e.g., infant mortality, asthma, or cardiovascular, stroke or HIV) is incorporated in the composite Index. These are what we want to view in terms of the Index. Upon maximal refinement of the HOI, we can then explore how these health-based indicators relate to the broader community conditions.

**Figure 2:**
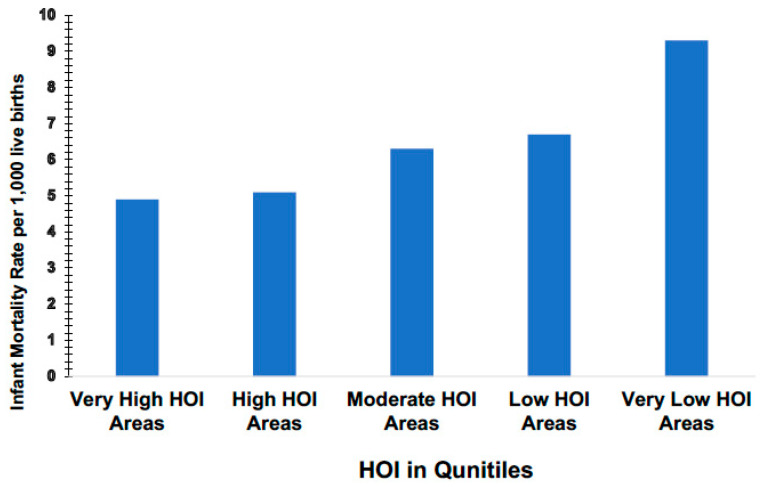
Health Opportunity Index Scaled into Quintiles against infant mortality to show the relationship. Data shows that the infant mortality rate in “Very High Opportunity Areas” on average is 47.3% lower than the rate in “Very Low Opportunity Areas”.

The state of Virginia is our reference point for the maturation of the HOI and in Virginia, the aforementioned independent variables were analyzed to determine which of these variables were associated with birth outcomes. As can be seen in Figure 2 below, on a statewide level, infant mortality per 1000 live births increases with HOI quintile to demonstrate that HOI variables were in fact, associated with low birth weight infants and infant mortality. We can conclude that residents of very low HOI quintiles are more likely to experience infant deaths by a striking 48.9% as compared to residents of very high HOI quintiles. This striking disparity and trajectory by HOI quintile are also true for diabetes hospitalization.

**Post Processing of Data via Principal Component Analysis (PCA):** As described above, the 13-indicators for the HOI were carefully chosen as core variables to provide a broad aerial view of an “opportunity structure” that can be easily and intuitively recognized by most community residents. These indicators therefore circumscribe what residents in a particular area have to navigate to accomplish their normal daily life tasks. We chose to apply principal component analysis (PCA) to reduce the noise and the dimension of the data structure, as recently discussed by Kahlia et al., (2020) [21]. The 13-place based indicators were normalized (Z scores), combined, and weighted using PCA to discern local patterns in the data at the census tract level. The use of PCA is greatest in scenarios where there are a large number of variables being considered in answering a question. In these situations, it is difficult to partition and understand how each variable is interacting [22]. Engaging in dimensional reduction reduces the amount of interactions between variables and PCA achieves this through feature extraction by combining variables in specific ways. The methodology prioritizes critical parts of the variables while leaving out the less important aspects of the variables.

Therefore, PCA was used in the present study to find the appropriate latent variables that might measure the landscape of an area. To maximize all indicators, the 13-indices were standardized into Z-Score and rotated using Varimax with Kaiser Normalization method to retain four components based on an eigenvalue cut-off of 1 or more. This process means that, if the eigenvalue is greater than 1, it indicates that that component explains more variance than a single variable because the sum of the eigenvalues equals the number of variables in the model. Each component was weighted using the proportion of their variance explained in the model as a percent of the cumulative variance and summed (weighted sum) to compute the composite index for each census tract. Table 1 demonstrates for example, that the variance of first component was 35.868% (Unweighted) while the cumulative variance for the four retained components summed to 73.443%. The first component was then weighted as 49.2% (35.868/72.857) *100. The process was repeated for all the three remaining components and were subsequently summed to obtain the composite index, known as the Health Opportunity Index (HOI).

## 4. Results

The HOI analysis results are presented in Table 2 (below) using a census tract in Cuyahoga County as an example. The analysis includes a number of data points which can be assessed. The primary data point is the HOI composite score. Step 1: HOI composite score and profile scores range from 0 to 1. The closer the score is to zero, the lower the chances for residents in the census tract to experience opportunity for good health. Conversely, the closer the score is to 1, the greater the chances that residents will experience high opportunities for good health. The public health professional can gauge health opportunity by looking at the Quintile. Quintile 1 reflects low health opportunity. Quintile 5 reflects high health opportunity. Life expectancy can also help gauge health opportunity. Step 2: Once overall health opportunity is determined; the public health professional can search for the social determinants that drive health opportunity. This requires an analysis of each of the Profile scores (Environmental, Consumer, Economic, and Population Mobility) to discern the profile with the lowest score. Step 3: Based on the Profile with the lowest score, (in this case, Consumer Profile) the public health professional would select the social determinant(s) for that profile with the lowest score (in this case, Food Access). Step 4: A public health professional might use other available data for the convergence analysis (see Methodology section). The convergence analysis reveals that there are six (6) health outcomes that are simultaneously at the worst levels in this census tract. Step 5: Interpret/Summarize Findings (2016 Data): Census Tract 39035118602 has overall low health opportunity for the residents to achieve good health. These sub-optimal outcomes include Poor Mental Health, Diabetes, Stroke, Coronary Heart Disease, Asthma, and High Blood Pressure. The 2017 500 Cities data also reflects that chronic obstructive pulmonary disease and kidney disease are at their worst levels. A deeper examination of the HOI data reveals that the social determinants of the Consumer Profile drive the observed poor health opportunity. A closer examination of the Consumer Profile reveals that Food Access is a major factor contributing factor to poor health opportunity in this census tract. As a result, the public health professional might recommend that interventions to improve overall health opportunity and disparate health conditions must take into account Food Access.

As mentioned above, the composite index score serves to provide an overall indication for the health of a particular community. The relative profiles of a particular tract can then be examined to explain what variables are making the greatest contribution to a low (or high) composite score. By analyzing the various profile scores, the low numbers are indicative of the primary drivers of poor health opportunity in the community. By extrapolating further, the specific index within the profile can be identified as the primary driver of poor health opportunity in the community. The HOI analysis report in Table 2 provides an example of the composite score for a census tract in Cuyahoga county. The 0.041 score is reflective of a low health opportunity for the census tract. This is reinforced by the score’s inclusion in the first quintile. By examining the four profiles, the consumer profile is shown to be the primary driver of low health opportunity with a score of 0.095. Further, food access appears to be the index that is driving the consumer profile.

**Figure 3:**
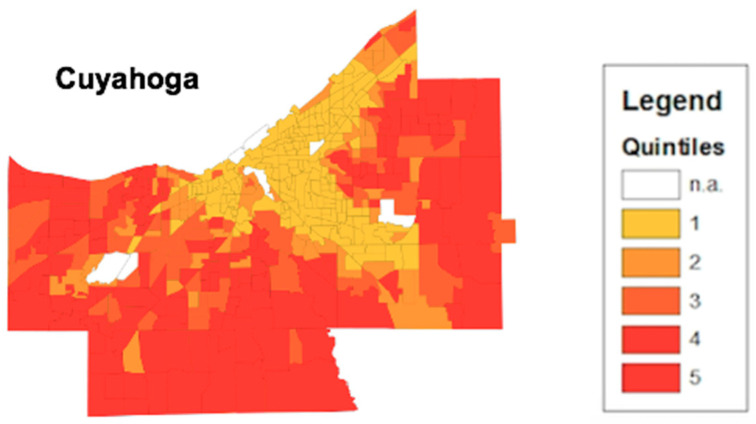
HOI Composite Score map from Cuyahoga County. The thematic map displays census tracts in Cuyahoga County in northeast Ohio. The census tracts are color coordinated based on what quintile their HOI composite score falls in. The lower HOI Composite scores fall in the first quintile while the higher HOI scores fall in the fifth quintile.

**Figure 4:**
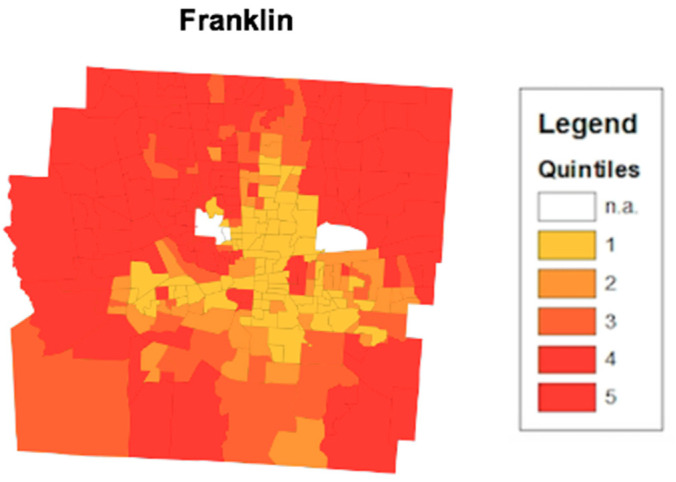
HOI Composite score map from Franklin County. The thematic map displays census tracts in Franklin County in central Ohio. The census tracts are color coordinated based on what quintile their HOI composite score falls in. The lower HOI Composite scores fall in the first quintile while the higher HOI scores fall in the fifth quintile.

The thematic mapping of HOI composite scores provides a visual breakdown of health opportunity disparities across census tracts in an area. Figure 3 depicts an ArcGIS map with a shapefile containing composite scores from the Cuyahoga county area. Using the thematic map feature from ArcGIS, differing census tract composite scores can be highlighted by quintile. The closer the census tract is to yellow, the lower the health opportunity. Census tracts highlighted in red provide higher health opportunity. The concentration of yellow census tracts aligns with the metropolitan area of downtown Cleveland, Ohio. Figure 4 depicts the same HOI composite score but in the Franklin county area. The concentration of the yellow census tracts aligns with the metropolitan area of downtown Columbus, Ohio.

**Figure 5:**
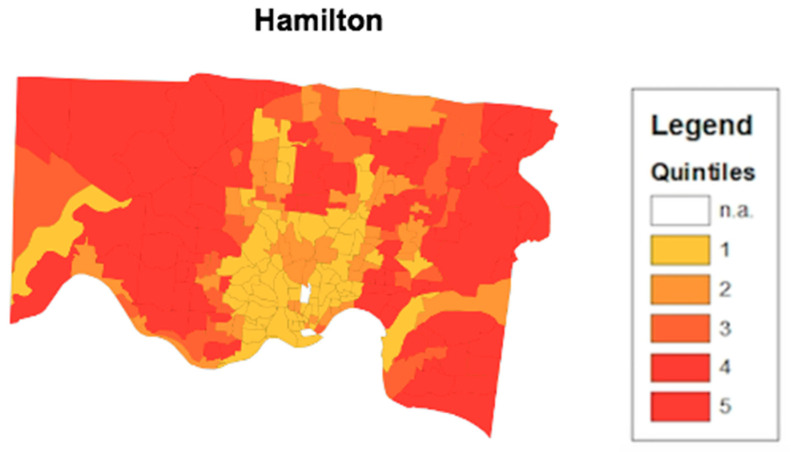
HOI Composite Score map from Hamilton County. The map displays census tracts in Hamilton County in southwest Ohio. The census tracts are color coordinated based on what quintile their HOI composite score falls in. The lower HOI Composite scores fall in the first quintile while the higher HOI scores fall in the fifth quintile.

**Figure 6:**
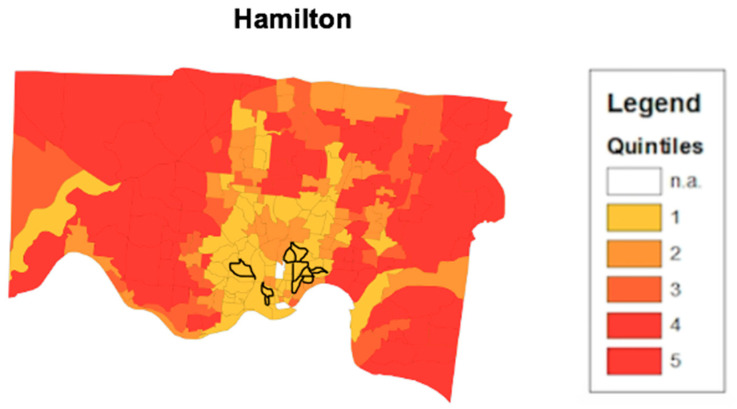
Disease Convergence as determined by HOI Composite score in Hamilton County. The census tracts outlined in black indicate the disease convergent census tracts. The indicated census tracts are areas where multiple diseases are occurring at high levels, simultaneously.

To determine the validity of the health opportunity index’s ability to predict high or low health opportunity, a disease convergence layer was added to the map. A disease convergent area is a census tract where multiple negative health outcomes simultaneously occur at their worst levels. Using the 500 Cities online database, crude prevalence rates for health outcomes such as coronary heart disease, COPD, asthma, chronic kidney disease, and diabetes were analyzed at the census tract level. Figure 5 shows a HOI composite score map for the Hamilton County area. Similar to the map from Figure 3 in Cuyahoga county, the census tracts with the lowest opportunity scores are highlighted in yellow and concentrated around the metropolitan area around downtown Cincinnati, Ohio. The Convergence Analysis for census tract FIPS Code 39035118602 revealed that there were six health conditions that simultaneously exist at their worst levels. They include poor mental health, diabetes, stroke, coronary heart disease, asthma, and high blood pressure. In Figure 6, the additional layer for disease convergence in Hamilton county Ohio has been added to the map as a cluster of outlined census tracts in the lowest scoring areas.

## 5. Discussion

The HOI can function as an essential tool in advancing health equity. It is important to note that the HOI tool is not meant to assess health outcome. The purpose of this tool is to provide public health practitioners, policy makers, and local organizations with a tool to assess SDH at the census tract level. The HOI can then inform the actions of policy makers and local communities who embark on the difficult work of improving the most serious health concerns of their communities by addressing the SDH that impacts health.

First, the HOI provides a method of comparing overall health opportunities of surrounding areas using the composite score. It is important to note that the HOI does not function as a health ranking. While the composite HOI score can be used for comparison of health opportunity among counties, its great advantage is its ability to highlight challenges and opportunities within the county and its census tracts. When applying and allocating funds for health-related grants, it is critical that all parties use a tool that is standardized to provide a common understanding of SDH which drive health outcomes.

The HOI also provides insight to similar challenges faced by different communities. The ability to highlight which census tracts have the lowest opportunities for optimal health can lead to specific policies, social, economic, environmental and structural changes to advance health equity. Further, breaking down the HOI by examining its subcomponents makes it possible to highlight which aspects of a community need the most attention to increase overall health. For example, in Table 2, the consumer profile has a score of 0.095 which is identified as the primary driver of low health opportunity. The food access index score of 0.063 signifies that the census block in Cuyahoga county suffers particularly from access to grocery stores, supermarkets and other suppliers of healthy food. With this information, the state’s public health department or community supporters could implement healthy food interventions to improve food access. This detailed process offers a more accurate method of addressing health equity issues.

The disease convergence layer is added over the HOI in order to provide better context of health. That is, to understand how different types of health disparities, at their worst levels, reflect low health opportunity and all of its complications. Figure 6 highlights 10 census tracts that are highlighted as convergence areas in Hamilton county. Each of the convergence area census tracts highlighted are in low health opportunity census tracts. The occurrence of multiple chronic health conditions simultaneously present at their worst levels in a single census tract indicate the influence of challenging SDH on these disparities. One of the shortcomings in public health practice is the proliferation of programmatic silos, which often address a particular health issue at any given time. Convergence analysis provides the opportunity for cross sector collaboration. In a census tract where diabetes, heart disease and hypertension all are occurring at their highest levels, health organizations with different programs can combine resources to address health conditions simultaneously.

Moreover, the HOI empowers collaborating organizations to be able to identify specific SDH to focus their collaborative interventions on. The HOI provides a large-scale image of an area’s health opportunity. Figure 3 highlights the census tracts that make up Cuyahoga county. The thematic map presented displays the concentration of low health opportunity census tracts closer to proximity to downtown Cleveland. Urbanization provides a number of factors that can drive the health opportunity of an area down such as overcrowding, air pollution, and high unemployment rates [23]. These factors provide a potential explanation of the differences in health opportunity between downtown Cleveland in Figure 3 and Columbus in Figure 4 and their surrounding suburban census tracts. Additionally, the thematic map allows stakeholders and policy makers to identify potential systematic issues that could be linked to low health opportunities. Demographic information on the low health opportunity census tracts are publicly available. For Figure 3, the converging point for the cluster of low health opportunity census tracts is Cleveland city, Ohio. According to the United States Census Bureau, the Cleveland city comprises approximately 25% of the Cuyahoga county population and is predominately African American (49.6%) [24]. Combining this knowledge with the HOI provides the potential for highly detailed and accommodating interventions in areas of the most need.

## 6. Conclusions

Our dashboard can be utilized by state and local health departments in municipalities across the US. Microsoft Access and ArcGIS Online can be used to link the HOI with Convergence Analysis to create reports and thematic maps to better visualize and contextualize both datasets to target vulnerable communities. In the present study, the use of 500 Cities data featuring the natural brakes classification method with seven (7) classes proved to be very useful, as was the 2013–2017 Vital Statistics data on prematurity, and the 2017 lead data using the equal ranges classification method with five classes (BRFSS 2017). Each dataset was selected based on the highest two classes that reflected the highest burden of disease. All data were imported into a MS Access database system. A query entitled “convergence” was created to create linking both dataset by the census tract FIPS code. A report was then created to identify census tracts that had at least four (4) of any of the conditions with a given census tract. Additionally, to identify census tracts for the convergence of different health issues, a filter tool in ArcGIS Online can be easily applied. By selecting the map layers with the appropriate data, it is possible to mimic the function of the MS Access query by using the filter for the highest rates of disease based on the natural breaks or equal range classes of the original dataset.

Relevant to the public health emergency that we are currently experiencing, the HOI provides an opportunity for municipalities to address the capacity of health systems by identifying areas where the availability of physicians are low using a provider-to-population ratio. Prior to the COVID-19 pandemic, the HOI was able to identify communities where access to health care was limited. This access, or lack thereof, would be expected to be further exacerbated and strained during a pandemic such as the current COVID-19 pandemic. As we have witnessed, as the pandemic has worsened across the United States, we continue to observe an increase in the population in need of health care as cases of COVID-19 continue to rise. Further, it is now apparent that these very low and low HOI areas are synonymous with the census tracts that are most disproportionately impacted in the COVID-19 pandemic. Therefore, we put forth the proposition that this novel tool will allow local, state, regional and national agencies to respond to COVID-19 impacted areas and other chronic health concerns due to its ability to predict and identify communities were the need for a robust response is likely to be the greatest.

## 7. Limitations

The HOI utilizes data sets from a variety of sources. For urban areas, often times this information is more readily available and accessible. However, for rural areas, the inability to access these data could prove difficult to incorporate the HOI evenly across an entire state. The HOI is still in its early stages of implementation so, Cleveland, Columbus, and Cincinnati are the initial major cities that have been HOI mapped across census tracts. Additionally, the HOI is created on a state by state basis. The index is built according to available datasets at the census tract level for that particular state, including variables that stakeholders and health departments identify as important and actionable. If the variables can be linked to health outcomes through academic literature, then they will serve as the framework for that area’s HOI. The HOI for the state of Ohio does not contain the same indices as the HOI for the state of Virginia. For this reason, cross state comparisons would prove to be difficult. Hopefully, nationwide adoption of the HOI would facilitate the creation of a nationwide baseline HOI.

Finally, the HOI is a measure of access or availability of opportunity within a community. This tool does not measure utilization or individual level barriers but rather assesses opportunity at the community level. Likewise, the HOI is not designed to address the sociocultural, organizational, geographic, and gender-related barriers in health care. Again, the purpose of the HOI tool is to move beyond the health care setting and examine the social determinants of health that impact health outcomes. While health care is an important determinant, the tool is aimed at assessing access to healthcare based on the availability of physicians and that segment of the population that is uninsured. Therefore, these issues are limitations of the HOI tool.

## 8. Future Directions

The differences in opportunity to achieve optimal health among communities around the state of Ohio further exasperate the disparities in health between these communities. The HOI is a novel tool capable of aiding public health officials and local stakeholders alike in identifying the differences in health opportunity. Conditions that drive poor health are extremely complex, exemplified by the possibility of two adjacent census tracts having low HOI scores caused by different SDH. Without having tools such as the HOI to understand which specific SDH drive poor health opportunity, it is highly likely that the wrong interventions will be applied, which will negatively impact efforts to advance health equity. It is not sufficient to just identify the differences, but understanding what factors are potentially driving down health are necessary for impactful health interventions. Using geospatial mapping tools such as ArcGIS, it is possible to create interactive tools that facilitate collaborative efforts to address the SDH on many different levels to improve impact disparate chronic health outcomes in census tracts. As communities continue to diversify, so do their health concerns and troubles. Addressing these growing concerns will require a more detailed intervention methodology by public health officials [25]. The implementation of the HOI presents a new tool to assist in this process. The fact that 12- of the 13 indices in the HOI are related to socioeconomic factors, its use in conjunction with the Public Health Exposome framework presents an exciting future for environmental health science [26]. The recently described Public Health Exposome framework can model the relationships between environmental and socio-demographic variables and health outcomes [27]. We have used the exposome framework to reveal latent associations between chemical stressors and low birth weights and pre-term births, both which have high prevalence rates in Ohio. Both the Public Health Exposome and the HOI incorporate datasets from a variety of locations to present a geographical approach to combating health disparities. Parallel use of these frameworks may prove to be beneficial to public health officials and local stakeholders alike.

In combination with the Public Health Exposome, the question as to how chemical stressors such as components of ambient pollution negatively impact vulnerable populations to result in premature death, including ischemic cardiovascular disease, stroke, respiratory infections, chronic obstructive pulmonary disease, and lung cancer might be more appropriately addressed [28,29,30,31,32]. Using the Public Health Exposome [33,34,35] and HOI analytics to integrate public health with exposure science and population-based epidemiological designs will likely provide novel and innovative theories and interventions to address disparate health outcomes. A recent study used data from the Global Burden of Disease 2016 study to address the question: “How much does exposure to chemical stressor (as PM_2.5_ air pollution) shorten human life expectancy around the world?” The study used a prior dataset that evaluated chemical stressor levels in 2000 and 2007 from 585 US counties and effects on life expectancy. The analysis revealed that in 2016, chemical stressor exposure reduced average global life expectancy at birth by ∼1 year with reductions of ∼1.2–1.9 years in polluted countries of Asia and Africa. A major conclusion from that study was; if exposure to chemical stressors in all countries met the World Health Organization Air Quality Guideline (10 μg/m^3^), the estimated life expectancy would increase by a population-weighted median of 0.6 year (interquartile range of 0.2–1.0 year), a benefit of a magnitude similar to that for eradicating lung and breast cancer [36]. This study and the many studies conducted prior to this most recent report serve to illustrate how mortality from exposure to airborne chemical stressors as environmental contaminants substantially reduces human longevity.

## Figures and Tables

**Table 1:** Explanation of the Total Variance in Derivation of Health Opportunity Index. The first component, PC1 explained 35.878% of the total variance while the cumulative variance of the four retained components summed to 72.857% based on the eigenvalue (1 or more).

Component	Initial Eigenvalues			Extraction Sums of Squared Loadings			Rotation Sums of Squared Loadings		
	Total	% of Variance	Cumulative %	Total	% of Variance	Cumulative %	Total	% of Variance	Cumulative %
**Component 1**	**4.664**	**35.878**	**35.878**	**4.664**	**35.878**	**35.878**	**4.306**	**33.124**	**33.124**
**Component 2**	**2.632**	**20.248**	**56.126**	**2.632**	**20.248**	**56.126**	**2.291**	**17.626**	**50.750**
**Component 3**	**1.148**	**8.831**	**64.957**	**1.148**	**8.831**	**64.957**	**1.719**	**13.226**	**63.977**
**Component 4**	**1.027**	**7.900**	**72.857**	**1.027**	**7.900**	**72.857**	**1.155**	**8.881**	**72.857**
Component 5	0.763	5.871	78.729						
Component 6	0.621	4.776	83.505						
Component 7	0.555	4.271	87.776						
Component 8	0.398	3.061	90.837						
Component 9	0.389	2.990	93.827						
Component 10	0.255	1.964	95.790						
Component 11	0.215	1.652	97.442						
Component 12	0.181	1.390	98.832						
Component 13	0.152	1.168	100.000						

1. Extraction Method: Principal Component Analysis. 2. The table above shows the total variance explained and based on the eigenvalue (1 or more), four components were retained.

**Table 2:** Health Opportunity Index score card. See text for details. The score card displays the composite, profile and index score from the HOI the scores range from 0 to 1. The closer the number is to 0, the more responsible the score is for driving low health opportunity.

Health Opportunity Index Score Card		Census Tract FIPS Code: 39035118602				
Composite Index	Environmental Profile		Consumer Profile	Economic Profile		Population Mobility Profile
0.041	0.74		0.095	0.141		0.892
	*Affordability*		*Education*	*Job Participation*		*Population Churning*
	0.862		0.35	0.341		0.914
**Quintile**	*Health Care Access*		*Food Access*	*Segregation*		
1	0.593		0.063	0.055		
	*Walkability*		*Material Deprivation*	*Income Inequality*		
**Life Expectancy**	0.718		0.176	0.352		
70.1	*Employment*					
	0.891					
	*Population Density*					
	0.851					
	*Environmental Quality*					
	0.664					
**Convergence Analysis**			**6** Health outcome(s) simultaneously at their worst levels:			
			*Poor mental health* *Diabetes* *Stroke* *Coronary Heart Disease* Asthma High Blood Pressure			

## Abbreviations

Health Opportunity Index, HOI; Public Health Exposome, PHE; Social Determinants of Health, SDH; Public Participatory Geographical Information Systems, PPGIS; Principal Component Analysis, PCA; SARS-CoV, Corona Virus Disease 19, COVID-19, Ohio Department of Health, ODH; US Department of Agriculture, USDA.
